# Fusogenic Coiled-Coil Peptides Enhance Lipid Nanoparticle-Mediated mRNA Delivery upon Intramyocardial Administration

**DOI:** 10.1021/acsnano.3c05341

**Published:** 2023-11-20

**Authors:** Ye Zeng, Mariona Estapé Senti, M. Clara I. Labonia, Panagiota Papadopoulou, Maike A. D. Brans, Inge Dokter, Marcel H. Fens, Alain van Mil, Joost P. G. Sluijter, Raymond M. Schiffelers, Pieter Vader, Alexander Kros

**Affiliations:** † Department of Supramolecular & Biomaterials Chemistry, Leiden Institute of Chemistry, Leiden University, 2333 CC Leiden, The Netherlands; ‡ CDL Research, University Medical Center Utrecht, 3584 CX Utrecht, The Netherlands; § Department of Cardiology, Laboratory of Experimental Cardiology, University Medical Center Utrecht, 3584 CX Utrecht, The Netherlands; ⊥ Regenerative Medicine Center Utrecht, University Utrecht, University Medical Center Utrecht, 3584 CT Utrecht, The Netherlands; ¶ Department of Pharmaceutics, Utrecht Institute for Pharmaceutical Sciences, Utrecht University, 3584 CS Utrecht, The Netherlands

**Keywords:** intramyocardial delivery, fusogenic coiled-coil, lipid nanoparticles, mRNA delivery, iPSC-CM

## Abstract

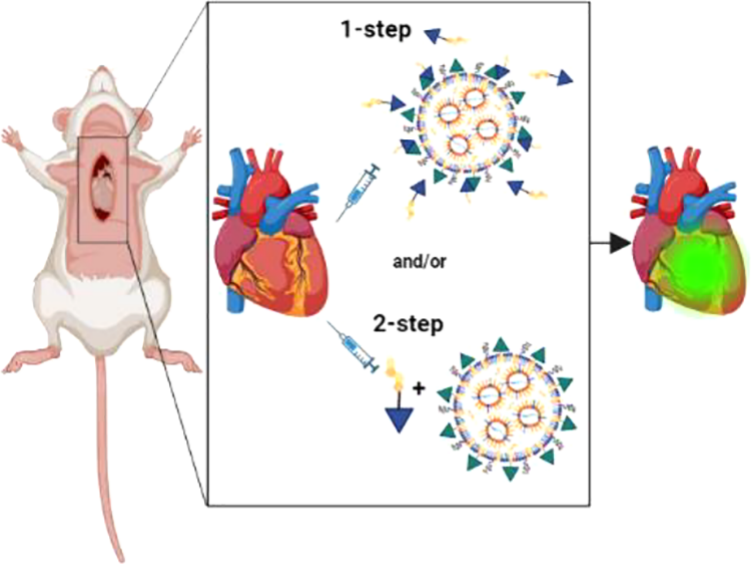

Heart failure is a serious condition that results from the extensive loss of specialized cardiac muscle cells called cardiomyocytes (CMs), typically caused by myocardial infarction (MI). Messenger RNA (mRNA) therapeutics are emerging as a very promising gene medicine for regenerative cardiac therapy. To date, lipid nanoparticles (LNPs) represent the most clinically advanced mRNA delivery platform. Yet, their delivery efficiency has been limited by their endosomal entrapment after endocytosis. Previously, we demonstrated that a pair of complementary coiled-coil peptides (CPE4/CPK4) triggered efficient fusion between liposomes and cells, bypassing endosomal entrapment and resulting in efficient drug delivery. Here, we modified mRNA-LNPs with the fusogenic coiled-coil peptides and demonstrated efficient mRNA delivery to difficult-to-transfect induced pluripotent stem-cell-derived cardiomyocytes (iPSC-CMs). As proof of *in vivo* applicability of these fusogenic LNPs, local administration via intramyocardial injection led to significantly enhanced mRNA delivery and concomitant protein expression. This represents the successful application of the fusogenic coiled-coil peptides to improve mRNA-LNPs transfection in the heart and provides the potential for the advanced development of effective regenerative therapies for heart failure.

## INTRODUCTION

Heart failure is a leading global cause of morbidity and mortality, largely due to the extensive loss of cardiomyocytes (i.e., heart muscle cells) resulting from acute or chronic ischemia.^1^ Unfortunately, the limited regenerative capacity of the adult mammalian heart after myocardial infarction, makes this loss irreversible, ultimately leading to pump dysfunction and heart failure.^2^ Although medical and device-based treatments can alleviate the symptoms, they fail to regenerate functional myocardium.^3^ Therefore, effective delivery systems capable of inducing cardiac repair and facilitating cardiomyocyte regeneration to rescue ischemic myocardium are urgently required.^4^

For *ex vivo* cardiomyocytes studies, primary cardiomyocytes are difficult to isolate and have a short lifespan, and many techniques have been adopted to obtain reliable sources of human cardiomyocytes, including bone marrow-derived, embryonic stem cells (ESCs), and induced pluripotent stem cells (iPSCs).^5,6^ Among these, iPSC-derived cardiomyocytes (iPSC-CMs) are the most promising cell source for cardiac repair research, as they can proliferate indefinitely and differentiate into cardiac lineages such as smooth muscle cells, endothelial cells, and cardiac progenitors.^7−9^ Previous studies have reported that cardiac expression of specific proteins, such as Yes-associated protein (YAP), VEGF, or angiopoietin-1 (Ang1), in adult mice, could enhance cardiomyocyte survival or proliferation, stimulate angiogenesis and improve cardiac function after MI.^10−15^ However, the continuous expression of these proteins may result in uncontrolled cardiac repair.^16^ To address this issue, mRNA (mRNA) therapeutics may be employed, as they possess temporary activity due to their natural degradation, providing temporal control over protein expression to stimulate regeneration while avoiding uncontrolled long-term growth. Indeed, mRNA therapeutics have already been shown to induce vascular regeneration after myocardial infarction *in vitro* and *in vivo*.^17−20^ However, delivering relevant therapeutic doses of these highly charged, immunogenic, and membrane-impermeable mRNA molecules to cardiac cells *in vivo* remains a major challenge.

There are several biomaterial designs and modifications that show promise for the safe delivery of mRNA, including lipids, lipid-like materials, polymers, and inorganic nanoparticles.^21^ Currently, lipid nanoparticles (LNPs) are the state-of-the-art vector for packaging, protecting, and releasing mRNA molecules within cells, as evidenced by the success of two COVID-19 mRNA-LNP vaccine formulations that received FDA approval in 2020.^20−24^ LNPs are typically composed of four lipid components—an ionizable lipid, cholesterol, a helper lipid, and a poly(ethylene glycol)-lipid conjugate, which can be varied to produce distinct formulations with desired physicochemical properties.^25−29^ However, the efficacy of LNPs in transfecting cells is limited by their ability to induce endo/lysosomal escape since they enter cells through endocytosis and are subjected to endolysosomal trafficking.^30^ Typically, less than 5% of the intracellularly delivered dose of mRNA by LNPs is able to reach the cytoplasm.^31^ Therefore, enhancing endosomal escape or omitting endosomal pathways by the fusion of LNPs with the cell membrane could significantly increase the clinical efficacy of RNA-based therapeutics.

**Scheme 1:**
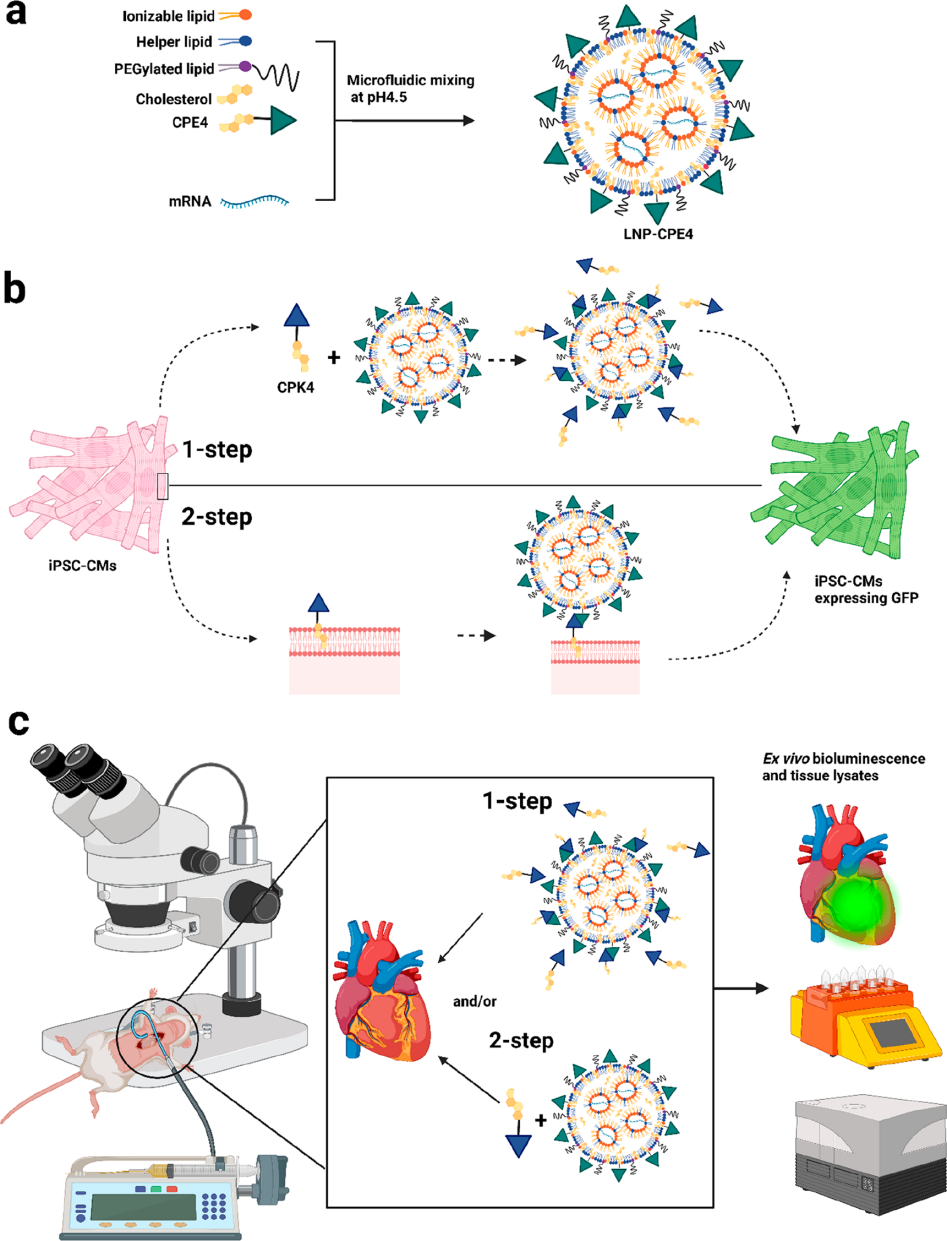
Overview of LNP Formulation and Delivery *in Vitro* and *in Vivo*. (a) Schematic Illustration of mRNA Encapsulating LNP-CPE4. (b) Fusogenic Coiled-Coil Peptide Modified Lipid Nanoparticles (LNPs) for EGFP-mRNA delivery in iPSC-CMs. (c) Schematic Illustration of Intramyocardial Administration of LNPs Encapasulating Luciferase-mRNA In the 1-step protocol, CPK4 and LNP-CPE4 are premixed and added to the cells. In the 2-step protocol, cells were first pretreated with CPK4 before incubation with LNP-CPE4.

Previously, we reported that efficient fusion of liposomes with cells *in vitro* and *in vivo* can be achieved using the complementary pair of coiled-coil lipopeptides CPE4/CPK4.^32−35^ These synthetic fusogens are composed of coiled-coil peptides conjugated to a cholesterol moiety via a polyethylene glycol (PEG) spacer ensuring readily incorporation in lipid membranes and nanoparticles.^36−40^ Fusion-mediated delivery of low molecular weight drugs into cells required a 2-step incubation protocol: cells were first pretreated with CPK4 and subsequently incubated with CPE4 functionalized liposomes carrying the drug of interest. In the current study, we investigated for the unexplored potential of using the complementary coiled-coil peptide pair CPE4/CPK4 for enhanced LNP-mediated mRNA delivery. We developed a 1-step incubation protocol compatible with *in vivo* applications (Scheme 1). For this, micellar CPK4 and CPE4-modified LNPs were briefly premixed before addition to cells including iPSC-CMs or *in vivo* administration. Fusogenic coiled-coil-peptide-mediated mRNA-LNPs delivery was evaluated *in vitro* and *in vivo* after local intramyocardial administration, and the applicability for various clinically approved LNPs was investigated.

## RESULTS AND DISCUSSION

### LNP Design, Formulation, and Characterization

We formulated various clinically approved LNP formulations encapsulating mRNA (encoding for enhanced green fluorescent protein, EGFP), and modified these LNPs with CPE4 (Scheme 1a, Scheme S1, Table S1). Physicochemical characterization of the LNPs using dynamic light scattering (DLS) and zeta-potential measurements showed that lipopeptide CPE4 modified LNPs showed a close hydrodynamic diameter with around 10 nm increase, similar polydispersity and zeta potential (near-neutral) with unmodified LNPs (Table S2). The mRNA encapsulation efficiency was slightly reduced after CPE4 modification, which was possibly due to negative charges of CPE4 impeding mRNA encapsulation. This showed that clinically approved LNP formulations can be modified with lipidated coiled-coil peptide (1 mol%) without altering the physicochemical properties.

**Figure 1:**
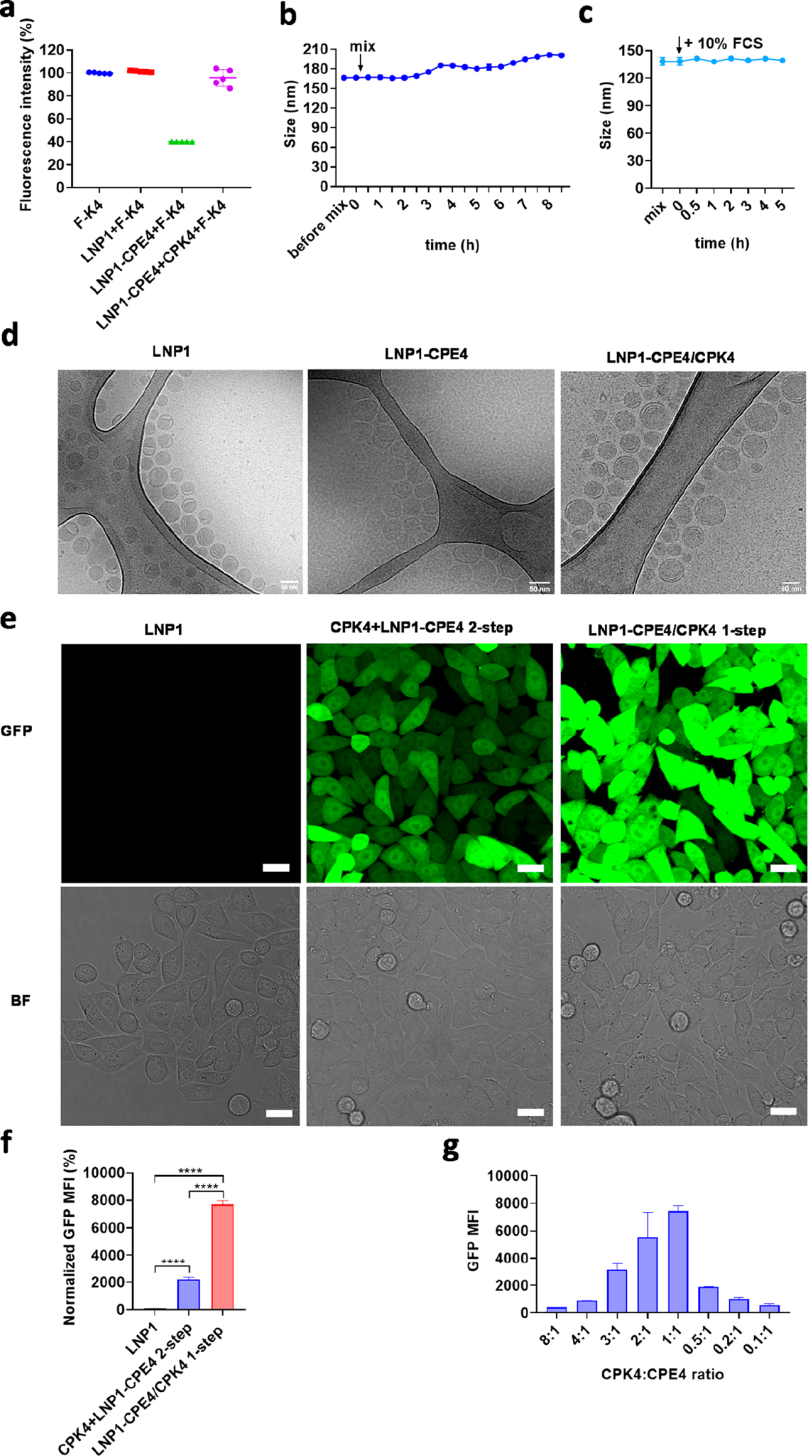
Premixing of CPK4 and CPE4 modified LNPs results in efficient transfection in cells. (a) Fluorescence intensity changes of fluorescein-labeled K4 peptide after addition to LNPs. (mean ± s.d., *n* = 5). (b) Hydrodynamic diameter changes over time after mixing LNP1-CPE4 with CPK4. (mean ± s.d., *n* = 3). (c) Hydrodynamic diameter changes over time after adding 10% FCS to the mixture of LNP1-CPE4 and CPK4. The nanoparticle diameter was monitored by DLS (mean ± s.d., *n* = 3). (d) Representative cryo-EM images of LNP1, and coiled-coil peptide modified LNP1-CPE4 before and after mixing with the complimentary peptide CPK4. The scale bar represents 50 nm. (e) Confocal microscopy images of EGFP-mRNA transfection of LNPs. CPK4+LNP1-CPE4 2-step: HeLa cells were pretreated with CPK4 (10 μM) for 2 h; then, the medium was removed, and LNP1-CPE4 was added (EGFP-mRNA, 1 μg/mL) and incubated for 24 h before imaging. LNP1-CPE4/CPK4 1-step: medium containing CPK4 (10 μM) and LNP1-CPE4 (EGFP-mRNA, 1 μg/mL) was added to HeLa cells and incubated for 24 h before imaging. GFP: green fluorescent protein; BF: bright field; scale bar is 20 μm. (f) Flow cytometry measurements of GFP expression intensity (GFP MFI) of LNPs. MFI was normalized to the LNP1 group. (mean ± s.d., *n* = 3, ****, *P* < 0.0001, ***, *P* < 0.001, **, *P* < 0.01, *, *P* < 0.05, ns, no significant difference). (g) CPK4:CPE4 ratio optimization upon a 1-step incubation protocol in HeLa cells (EGFP-mRNA, 1 μg/mL). (mean ± s.d., *n* = 3)

Our LNP formulations contain PEG2K chains (the contour length is 12.7 nm), which may hinder the binding of CPK4 to CPE4 (the contour length is 12.8 nm). As efficient mRNA delivery requires coiled-coil formation, we tested whether CPE4 in LNPs is accessible to CPK4 binding. Hereto, we developed a fluorescence assay to monitor the binding affinity between CPE4 and fluorescein-labeled K4 peptide (F-K4) by measuring the fluorescence intensity changes after centrifugation (Figure 1a). Free F-K4 peptide was used as a control, showing 100% fluorescence intensity and indicating no binding. As expected, when F-K4 was added to unmodified LNP1, the fluorescence intensity was similar to that of free F-K4, demonstrating that free F-K4 failed to interact with unmodified LNP1 in the absence of CPE4. In contrast, when F-K4 was added to LNP1-CPE4, the fluorescence intensity showed a significant reduction to 40%, indicating that F-K4 successfully binds to CPE4 on the LNP surface. When F-K4 was added to a premixture of LNP1-CPE4 and CPK4, the fluorescence intensity was 96%, similar to that of free F-K4, indicating that all CPE4 was already occupied by CPK4 via coiled-coil formation. Thus, our assay confirmed that even though the peptides may be partially buried in the PEG brush, they are still capable of forming coiled-coil structures.

### Incubation Protocol Influences mRNA Delivery and Protein Expression in Cells

Previously, we showed that coiled-coil mediated fusion significantly improves the delivery efficiency of liposomes in various cell lines.^32−35^ In these studies, we used a 2-step incubation protocol which requires the pretreatment of target cells with CPK4 before the addition of liposomes containing CPE4 (Scheme 1b). To enable the translation of this strategy toward (pre)clinical evaluation, we developed a 1-step approach where CPK4 and CPE4 modified LNPs were premixed before administration.

To ensure that the premixing of CPK4 and CPE4-modified LNPs did not cause significant aggregation, we measured the hydrodynamic diameters of modified LNPs over time using dynamic light scattering. Our observation showed only a slight size increase after 2.5 h, indicating that premixing CPK4 and LNP1-CPE4 did not induce aggregation in the buffer or the presence of 10% fetal calf serum (FCS) (Figure 1b, c).

We also investigated the lipid membrane rigidity of LNPs using a fluorescent probe TMA-DPH (Figure S1b, Supporting Information).^41,42^ This probe embedds in the outer leaflet of the bilayer, adopting a preferential orientation that alters its spatial anisotropy, with a decrease in anisotropy indicating a reduction in the overall organization of the bilayer structure.^43^ We utilized this probe to investigate whether lipid membranes of LNPs exhibit differences in rigidity after introduction of coiled-coil peptides. We tested the probe anisotropy by increasing the temperature from 20 to 80 °C. LNP1 showed the highest anisotropy throughout the entire tested range. CPE4 introduction slightly reduced the anisotropy of LNP1 and suggested a more disorganized bilayer structure. However, CPK4 addition to LNP1-CPE4 restored the anisotropy to some extent. Therefore, the introduction of a coiled-coil peptide did not significantly influence the fluidity of LNPs.

We then investigated the morphology of CPE4-modified LNPs in the presence or absence of complementary CPK4 using cryo-electron microscopy (cryo-TEM). Our findings revealed that the core of both LNPs displayed the characteristic mixture of amorphous, unilamellar, and polymorphic structures (Figure 1d),^43,44^ suggesting that the addition of CPE4 to LNPs does not change the LNP internal structure. In line with the dynamic light scattering data, CPE4-modified LNPs mixed with CPK4 did not induce changes in structure or induced aggregation. In summary, premixing CPE4-modified LNPs with micellar CPK4 does not negatively influence the stability of the nanoparticles, allowing for the use of a 1-step incubation protocol in future *in vivo* studies.

Next, we studied the mRNA delivery of LNPs in HeLa cells by confocal imaging and flow cytometry analysis (Figure 1e,f, Figure S1c, and Supporting Information). Our results showed that significantly higher GFP expression was obtained when the LNPs were modified with the fusogenic coiled-coil peptides. Surprisingly, the 1-step incubation protocol induced a stronger GFP expression compared to the 2-step incubation protocol, and the cholesterol-PEG linker of CPK4 was necessary to achieve high transfection. To optimize GFP mRNA delivery, we varied the CPK4:CPE4 ratio for the 1-step incubation protocol. Our findings revealed that the highest level of GFP expression was achieved when an equimolar ratio of CPK4:CPE4 was used (Figure 1g). Thus, the 1-step incubation protocol for preparing coiled-coil peptide modified LNPs is a viable mRNA delivery approach and the 1:1 ratio of CPK4:CPE4 is the optimal ratio to achieve maximal transfection enhancement. Consequently, we used this ratio in all subsequent experiments.

### Fusogenic Coiled-Coil Peptide Modified LNPs Enhance mRNA Transfection in iPSC-CMs *in Vitro*

**Figure 2:**
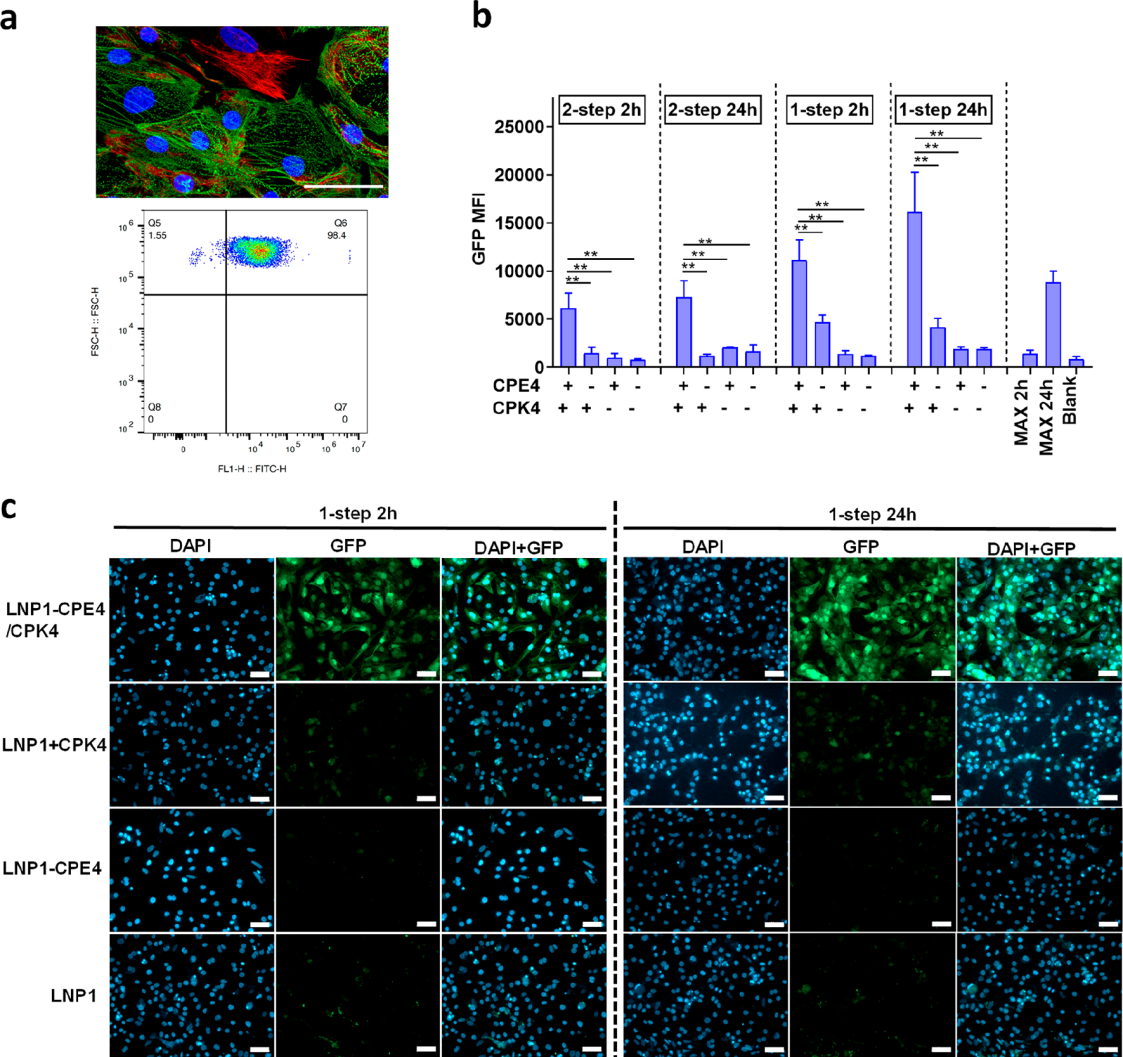
LNPs modified with fusogenic coiled-coil peptides display enhanced mRNA transfection efficiency in iPSC-CMs. (a) Characterization of iPSC-CMs: Characterization of iPSC-CMs: fluorescent immunostaining showing α-actinin positive iPSC-CMs (in green) and vimentin positive noncardiomyocytes (in red) present in 2D culture, and representative flow cytometry plot of percentage α-actinin positive cells after iPSC-CM differentiation showing high purity (98.4% α-actinin positive) of differentiation. (b) The transfection efficiency (GFP MFI) of LNPs in iPSC-CMs was measured by flow cytometry. 2-step 2h: iPSC-CMs were pretreated with CPK4 (10 μM) for 2 h and afterward incubated with LNP1-CPE4 (2 μg/mL) for 2 h. After 2 h, the supernatant was removed, and cells were cultured for another 24 h before flow cytometry measurements. 1-step 2h incubation: medium containing CPK4 and LNP1-CPE4 (2 μg/mL, CPK4/CPE4 = 5 μM) was added to the iPSC-CMs and incubated for 2 h. Next, the medium was removed, and cells were cultured for another 24 h before flow cytometry measurements. For the 2-step 24h and 1-step 24h groups: iPSC-CMs were incubated with LNPs for 24 h before measuring; all the other steps in the protocol remained the same. (****, *P* < 0.0001, ***, *P* < 0.001, **, *P* < 0.01, *, *P* < 0.05, ns, no significant difference) In all panels, error bars represent mean ± s.d. (*n* = 3). (c) The confocal microscopy images of LNP-mediated EGFP-mRNA transfection in iPSC-CM using a 1-step incubation protocol. Blue: DAPI; green: GFP, green fluorescent protein; scale bar represents 50 μm.

We next evaluated the *in vitro* transfection performance of LNPs modified with coiled-coil peptides on cardiac cells, i.e. induced pluripotent stem cells-derived cardiomyocytes (iPSC-CMs), which are arguably the most relevant model for preclinical drug screening due to their ability to model cardiac tissue.^45−47^ Prior to the screening, we characterized the iPSC-CMs by immunostaining for α-actinin by confocal imaging and flow cytometry measurement, which showed successful and uniform α-actinin staining and high differentiation purity (>98.4%) (Figure 2a).

We compared the transfection efficiency of LNPs at different time points (2h vs 24h) and using two incubation protocols. In line with the transfection results obtained in HeLa cells, the fusogenic peptide-modified LNPs showed highly efficient GFP expression in iPSC-CMs at both time points, with a higher transfection efficiency than that of the commercial mRNA transfection reagent Lipofectamine messengerMAX (Figure 2b). Consistent with the results obtained in HeLa cells, the 1-step incubation protocol was more effective than the 2-step protocol. Flow cytometry data showed that fusogenic coiled-coil peptides significantly increased iPSC-CM transfection, up to a 19-fold increase when using the one-step incubation protocol at 24h (Figure S2a), representing a significant improvement compared to state-of-the-art LNPs.

Confocal microscopy was also used to visualize GFP expression in iPSC-CMs following different LNP incubation protocols (Figure 2c, Figure S2b). Treatment with LNPs modified with fusogenic coiled-coil peptides resulted in strong and uniform GFP fluorescence in the majority of iPSC-CMs, irrespective of the incubation time (2h vs 24h). In contrast, transfection of the cells with unmodified LNPs yielded only weak GFP fluorescence. In summary, modifying mRNA-LNP formulations with fusogenic coiled-coil peptides significantly enhances mRNA transfection of difficult-to-transfect iPSC-CMs *in vitro*.

### Fusogenic Coiled-Coil Peptide Modified LNPs Display Enhanced Intramyocardial Transfection *in Vivo*

**Figure 3:**
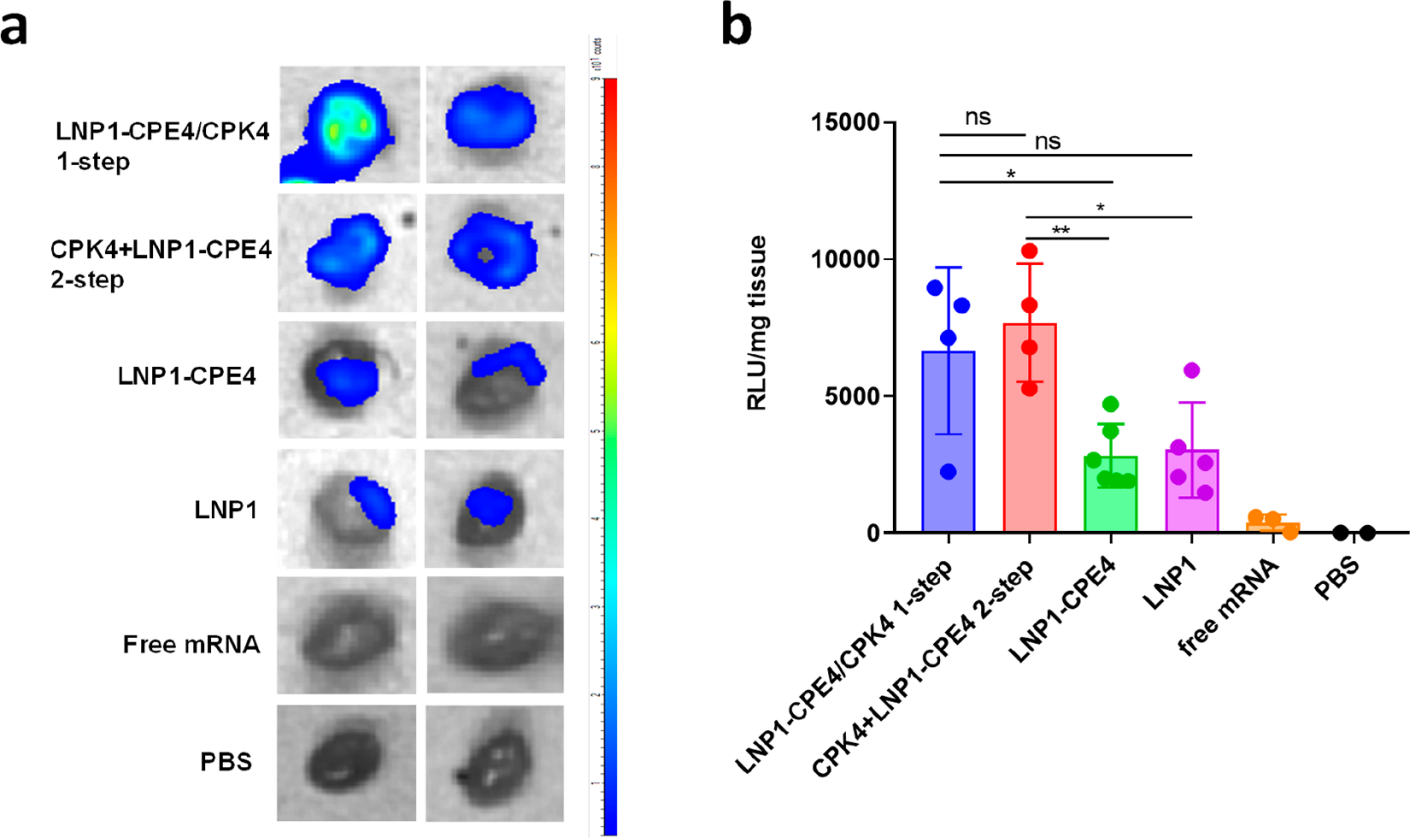
Coiled-coil fusogenic peptides enhance LNP-mediated mRNA delivery upon intramyocardial injection. Balb/c mice were intramyocardially injected with 5 μg (in 10 μL) of firefly luciferase mRNA encapsulated in (1) CPK4 and LNP1-CPE4 (final concentration of CPK4/CPE4, 125 μM), either premixed before injection or (2) injected in 2 sequential steps, (3) LNP1-CPE4 or (4) LNP1. As controls, free mRNA and PBS were also injected. Twenty-four h postadministration, organs were harvested, and the luminescence was measured by IVIS imaging. (a) Luminescence images of mice hearts. (b) Luciferase activity in heart lysates. Statistical significance was calculated with a one-way ANOVA (****, *P* < 0.0001, ***, *P* < 0.001, **, *P* < 0.01, *, *P* < 0.05, ns, no significant difference). Data are represented as the mean ± s.d. (*n* = 4 for LNP1-CPE4/CPK4 1-step and CPK4+LNP1-CPE4 2-step; *n* = 6 for LNP1-CPE4; *n* = 5 for LNP1; *n* = 3 for free mRNA; *n* = 2 for PBS.)

Initially, we conducted a pilot study to assess the efficacy of mRNA-LNPs administered intravenously in healthy mice. We encapsulated firefly luciferase mRNA in LNPs, injected them into the mice, and then measured luciferase activity in the organs *ex vivo* and lysates after 24 h (Figure S3a, b). As expected, luciferase expression in LNP-treated mice was mainly observed in the liver, with little to no signal in the heart. Modification of LNPs with coiled-coil peptides had no significant effect on the tissue distribution profile. Thus, to enable effective cardiac regenerative therapy and prevent disease progression after myocardial infarction, local administration may be crucial. Therefore, we evaluated the *in vivo* transfection performance of LNPs 24 h after intramyocardial injection and analyzed luciferase activity in mouse organs *ex vivo* and lysates (Scheme 1c). We observed that the mRNA transfection in the heart was significantly enhanced for the LNPs modified with fusogenic coiled-coil peptides, irrespective of the injection protocol used. This proved that 1-step injection is indeed possible without loss of functionality. Our state-of-the-art fusogenic LNPs outperformed naked mRNA administered in citrate buffer, which is the current state-of-the art for intramyocardial injections (Figure 3a, b).^12,47^ The liver remained the primary organ in which mRNA-LNPs were expressed after intramyocardial injections, followed by the spleen (Figure S4a, b). We expect that the performance of LNPs would improve in larger animals (including humans) since injecting materials into the beating heart of mice is technically challenging and results in significant direct flush-out into the bloodstream. Overall, our findings demonstrate that fusogenic coiled-coil modified LNPs can significantly enhance mRNA delivery *in vivo* upon intramyocardial injections.

We also assessed the *in vivo* safety profile of modified LNPs 24 h after administration by measuring the serum levels of liver enzymes including alkaline phosphatase (ALP), aspartate transaminase (AST), and alanine aminotransferase (ALT). For ALP, all groups displayed similar levels after both intravenous and intramyocardial injections (Figure S5a, d). For AST expression, no significant differences were found between groups except for a data point in the one-step injection of fusogenic coiled-coil modified LNPs that is almost 10× higher than all the other data points, which makes the one-way ANOVA analysis not significant (Figure S5b, e). Only for ALT, coiled-coil peptide modified LNPs induced higher levels than LNP1 after intravenous administration and higher levels than LNP1-CPE4 and LNP1 after intramyocardial administration (Figure S5c, f). We hypothesize that this toxicity could be due to the increased accumulation in the liver.

### Fusogenic Coiled-Coil Peptide Modified LNPs Also Display Enhanced mRNA Transfection When Using Other Ionizable Lipids

**Figure 4:**
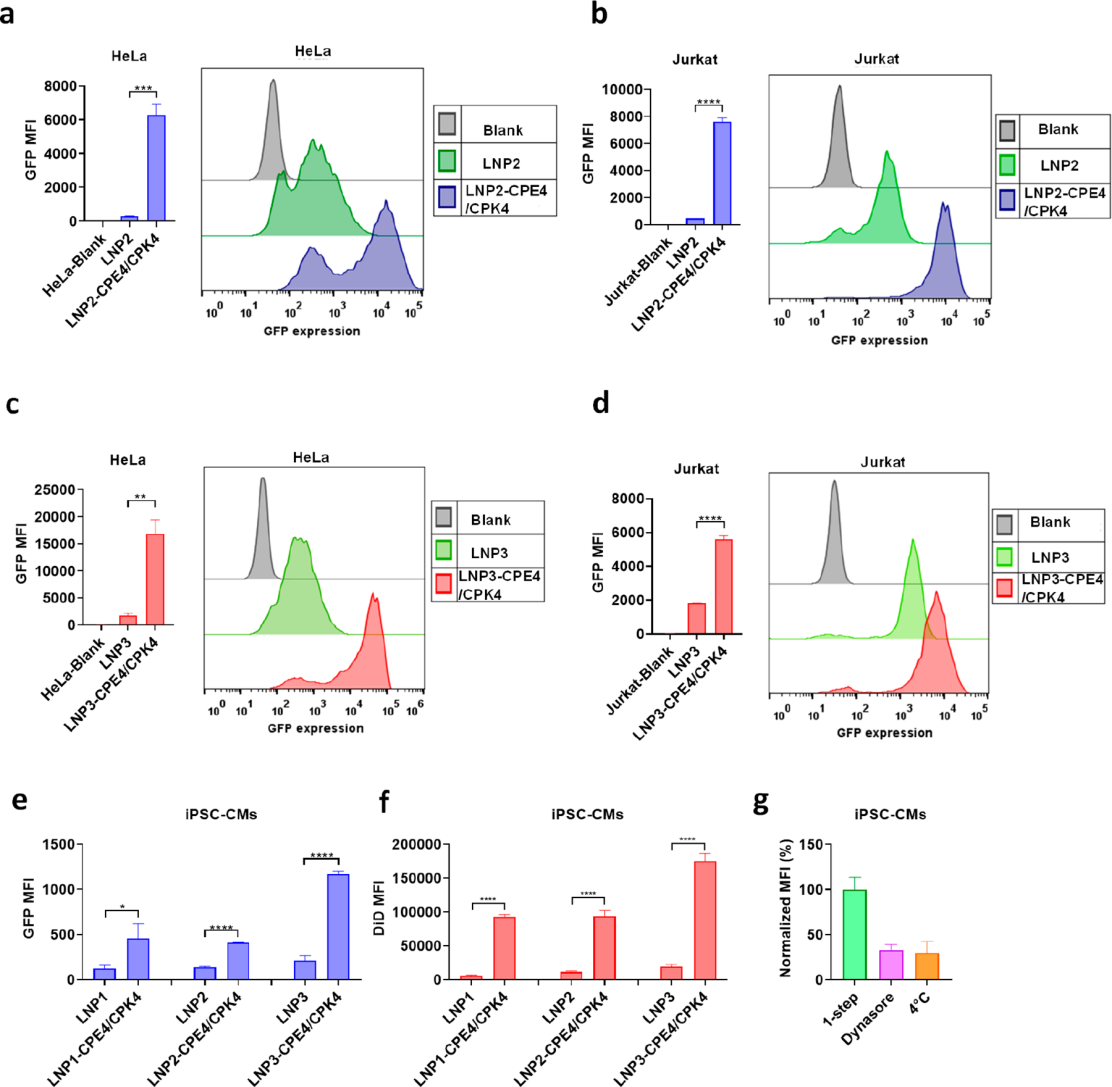
LNPs modified with fusogenic coiled-coil peptides display enhanced *in vitro* mRNA transfection efficiency for different ionizable lipids. (a–d) HeLa and Jurkat cells were transfected with LNPs containing different ionizable lipids (EGFP-mRNA, 1 μg/mL). LNP2 and LNP3 represent the Covid-19 mRNA-LNP formulations of Pfizer/BioNTech and Moderna, respectively. The transfection efficiency was analyzed 24 h after transfection utilizing flow cytometry (GFP MFI). (e) The transfection efficiency on iPSC-CMs of LNPs composed of different ionizable lipids was measured by flow cytometry after 24 h. Statistical significance was calculated by an unpaired student *t* test. (f) The cellular uptake efficiency of LNPs on iPSC-CMs using the 1-step incubation protocol and incubating for 24 h was measured by quantifying DiD fluorescence. 0.5 mol% of DiD was included in the lipid composition. Statistical significance was calculated by an unpaired student *t* test. (****, *P* < 0.0001, ***, *P* < 0.001, **, *P* < 0.01, *, *P* < 0.05, ns, no significant difference). In all panels, error bars represent mean ± s.d. (*n* = 3). (g) The cell uptake mechanism of fusogenic LNPs (LNP1) using 1-step incubation protocol on iPSC-CMs after 2 h incubation in the presence of endocytosis inhibitor or incubated at 4 °C. 0.5 mol% of DiD was included in the lipid composition and DiD intensity was normalized to 1-step of fusogenic LNPs in the absence of inhibitors. Error bars represent mean ± s.d. (*n* = 3).

LNP formulations contain ionizable lipids that can condense the genetic cargo and affect the transfection performance by influencing the endosomal escape efficiency.^48,49^ LNP1 modified with coiled-coil peptides displayed enhanced mRNA transfection both *in vitro* and *in vivo* upon local injection. This led us to investigate whether similar transfection improvements could be observed in other LNP formulations containing different ionizable lipids, such as ALC-0315 and SM-102, which are used in the two COVID-19 mRNA-LNP vaccine formulations.^21,23^ We observed improved GFP expression in HeLa and Jurkat cells when coiled-coil peptides were introduced to these LNP formulations using the 1-step incubation protocol (Figure 4a–d). These findings suggest that fusogenic coiled-coil peptides can enhance mRNA transfection across a broad range of LNPs.

We further evaluated the transfection performance of coiled-coil peptide-modified LNPs containing ALC-0315 and SM-102 lipids in iPSC-CMs using the one-step incubation protocol. Flow cytometry analysis of transfected iPSC-CMs revealed a significant improvement in GFP expression with the introduction of fusogenic coiled-coil peptides to LNPs compared to unmodified LNPs (Figure 4e). This further supports our findings that coiled-coil peptides can enhance the mRNA transfection of various LNP formulations on different cell lines, including iPSC-CMs. The improved transfection efficiency may be attributed to the enhanced cell uptake efficiency facilitated by coiled-coil peptide-modified LNPs using a 1-step incubation protocol. This is supported by the quantification of a significantly higher number of DiD-labeled LNPs in iPSC-CMs (Figure 4f).

We finally investigated the cell uptake mechanism of coiled-coil peptide modified LNPs on iPSC-CMs. After incubation with cellular endocytosis inhibitors, we employed flow cytometry to quantify the cellular uptake of DiD-labeled LNP1 as the representative LNP formulation. We showed that cellular uptake of both coiled-coiled peptide-modified LNP1 prepared by 1-step incubation as well as plain LNP1 could by blocked using dynasore and incubation at 4 °C, indicating that cell uptake was mainly mediated by active, (dynamin-dependent) endocytosis.^50−53^

## CONCLUSION

In our previous studies, we reported that modifying liposomes with coiled-coil lipopeptides resulted in improved delivery of liposomal cargo.^32−35^ In this study, we explored the use of heterodimeric coiled-coil lipopeptides (CPE4/CPK4) to modify mRNA-LNPs. Our earlier research involved a 2-step incubation process, where cells were first pretreated with CPK4 and then incubated with liposomes containing CPE4. To facilitate the translation of this technology to an *in vivo* setting, we further optimized the delivery protocol to a 1-step procedure by premixing CPK4 and CPE4-modified LNPs before adding them to cells or injecting them *in vivo*. We confirmed that the modification of LNPs with these lipopeptides did not affect their physicochemical properties. We evaluated this technology both *in vitro* and *in vivo*. *In vitro* studies revealed that coiled-coil lipopeptides significantly enhanced mRNA delivery of LNPs to iPSC-CMs, compared to that of unmodified LNPs. When we studied *in vivo* delivery efficiency of these nanoparticles, we observed that both 2-step and 1-step injection of LNPs modified with fusogenic coiled-coil peptides significantly improved mRNA transfection in the heart upon intramyocardial injection compared to other LNP groups. Moreover, we successfully applied the fusogenic coiled-coil peptides to two clinically approved COVID-19 mRNA-LNP vaccine formulations, demonstrating enhanced mRNA transfection for different cell lines including difficult-to-transfect cell lines such as Jurkat or iPSC-CMs. In summary, we show that modifying mRNA-LNPs with fusogenic coiled-coil peptides significantly enhances *in vitro* mRNA transfection of difficult-to-transfect cell lines and also improves *in vivo* mRNA transfection upon local intramyocardial injection. We envision that this technology holds great promise for the development of local mRNA-based therapies.

## METHODS

### Materials

Lipopeptides CPE4, CPK4, and fluorescently labeled K4 (fluorescein-K4) were synthesized as previously described.^32−35^ 1,2-Distearoyl-*sn*-glycero-3-phosphocholine (DSPC), 1,2-dimyristoyl-*rac*-glycero-3-methoxypolyethylene glycol-2000 (DMG-PEG2K) were purchased from Avanti Polar Lipids, DLin-MC3-DMA was purchased from Biorbyt (Cambridge, England), and cholesterol was purchased from Sigma-Aldrich. Triton X-100 was purchased from Acros Organics. Fetal calf serum was purchased from Sigma. 100k MWCO centrifugal filters (Amicon Ultra, Merck) were purchased from Sigma. Lipofectamine MessengerMAX was purchased from Thermofisher Scientific. QuantiT RiboGreen RNA Assay Kit was purchased from Life Technologies. Clean cap EGFP-mRNA was purchased from Trilink Biotechnology, and Luciferase-mRNA was a kind gift from Etherna (Belgium). The ionizable lipids ALC-0315 and SM-102 were synthesized according to the literature.^48,54^

HeLa and Jurkat cell lines purchased from ATCC were cultured according to the ATCC guidelines. DMEM and RPMI-1640 growth medium (Sigma Aldrich) containing sodium bicarbonate, without sodium pyruvate and HEPES, was supplemented with 10% fetal bovine serum (Sigma), 1% L-glutamine (Thermo Fisher Scientific) and 1% penicillin/streptomycin (Thermo Fisher Scientific), at 37 °C in the presence of 5% CO_2_. HeLa cells were cultured with DMEM medium, while Jurkat cells were cultured with RPMI-1640 medium. The fully characterized human iPSC line NP0144–41 was obtained from peripheral blood mononuclear cells using the Sendai virus reprogramming method at the University of Cologne.^55^ The line was deposited as cell line UKKi037-C at the European Bank for induced pluripotent Stem Cells (EBiSC, https://ebisc.org/) and is registered in the online registry for human PSC lines hPSCreg (https://hpscreg.eu/).

### Formulation and Characterization of Lipid Nanoparticles

Stock solutions of lipids and lipopeptides were mixed in a vial at the desired molar ratios (Scheme S1 and Table S1). Afterward, the solvents were evaporated under a nitrogen flow, and the residual solvent was removed *in vacuo* for at least 30 min. The lipid film was dissolved in absolute ethanol and used for the assembly. mRNA was diluted in 50 mM RNase-free citrate buffer (pH = 4). The solutions were loaded into two separate syringes and connected to a T-junction microfluidic mixer. The solutions were mixed in a 3:1 flow ratio of nucleic acid against lipids (1.5 mL/min for mRNA solution, 0.5 mL/min for lipids solution, N/P = 1/6). After mixing, the solution was directly loaded into a 20 kDa MWCO dialysis cassette (Slide-A-Lyzer, Thermo Scientific) and dialyzed against 1× PBS overnight. LNPs were concentrated using 100 kDa MWCO centrifugal filters and centrifuged for 1–2 h, at 4 °C and 5000 RCF.

The particle size and polydispersity index (PDI) of the mRNA-LNPs diluted in 1× PBS pH 7.4 were measured by dynamic light scattering (DLS) using Zetasizer Nano-S (Malvern Instruments Ltd., Worcestershire, UK) at 25 °C. The intramyocardial injection sample was prepared by concentrating LNPs to reach high mRNA concentration using Amicon Ultra-100k Centrifugal Filter Unit (Sigma), centrifuge in 4 °C, 5000 RCF, 1–2 h. Dynamic light scattering measurements (DLS) revealed that the observed hydrodynamic diameter of the LNP1-CPE4 was independent of concentration (Figure S1a, Supporting Information). The stability of the CPK4 and LNP1-CPE4 (concentrated *in vivo* sample, CPK4:CPE4 = 1:1) mixture was checked both in PBS and 10% FCS by measuring the hydrodynamic diameter changes in DLS. Zeta potential was measured by particle electrophoretic mobility using Zetasizer Nano-ZS (Malvern Instruments), using folded capillary zeta potential cells (DTS1070, Malvern), and LNPs were diluted in 0.1X PBS pH 7.4. Concentration and encapsulation of mRNA in LNPs were determined using the Quant-it RiboGreen RNA Assay. LNPs were diluted in 1X TE buffer with and without Triton X-100 (to induce LNP breaking), followed by the addition of the RiboGreen reagent diluted in 1x TE buffer. Standard curves were also prepared. RiboGreen fluorescence was measured using a TECAN Spark microplate reader (Männedorf, Switzerland). Encapsulation efficiency (EE) was calculated according to the following formula: EE (%) = [(Total mRNA – free mRNA)/Total mRNA] × 100.

The morphology of LNPs was analyzed by using cryogenic transmission electron microscopy (cryo-TEM). Vitrification of concentrated LNPs ([total lipid]∼10 mM) was performed using a Leica EM GP operating at 21 °C and 95% room humidity (RH). Sample suspensions were placed on glow-discharged 100 μm lacey carbon films supported by 200 mesh copper grids (Electron Microscopy Sciences). Optimal results were achieved using a 60 s preblot and a 1 s blot time. After vitrification, sample grids were maintained below −170 °C, and imaging was performed on a Talos with a LaB6 filament operating at 120 keV equipped with a Ceta camera. Images were acquired at a nominal underfocus of −2 to −4 μm (28.000× magnification) yielding a pixel size at the specimen of 3.4 Å. For cryo-TEM imaging, CPK4 was added to LNP1-CPE4 at a 1:1 ratio and incubated for 1 h before imaging.

For the binding assay, fluorescein-labeled K4 peptide was added to LNP1-CPE4 (CPK4:CPE4 = 1:1) and incubated at RT for 1–2 h, followed by centrifugation using Amicon Ultra-100k Centrifugal Filter Unit (Sigma) at 4 °C, 5000 RCF, 1–2 h. The solution in the lower part of the tube was collected, and fluorescence intensity was quantified with a Tecan plate reader (excitation wavelength: 480 nm; emission wavelength: 520 nm). The fluorescence intensity was normalized to that of free fluorescein-K4.

Membrane fluidity assay: TMA-DPH assay was performed as described elsewhere.^41,42^ TMA-DPH stock solution in ethanol was added to the final solutions to yield a 1:500 fluorophore to lipid mixture of LNP with pH 7 1× PBS buffer. The final total lipid concentration of LNP used for fluorescence measurements was 0.3 mM, and the fluorescent probe was 0.6 μM. The fluorescence anisotropy was recorded using a temperature-controlled automatic polarization setup at wavelengths 338 nm (excitation) and 446 nm (emission) (Steady state fluorescence spectrometer FS900). The temperature of the lipid dispersion was controlled to within 0.2 °C. The data presented are the averages of three independent measurements.

### Human iPSC Culture and Differentiation

All experiments were conducted according to the criteria of the code of proper use of human tissue in The Netherlands. Human iPSC lines were kindly provided by Tomo ari (University of Cologne, Germany). iPSCs were grown on growth factor-reduced Matrigel (Corning) and in Essential 8TM medium (Gibco A1517001), refreshed every other day. Cells were nonenzymatically passaged every 4–5 days with 0.5 × 10^–3^ M EDTA (Thermo Fisher Scientific). iPSCs differentiation to CMs was performed using a GiWi differentiation protocol adapted from Lin and colleagues.^56^ At day 0, starting with iPSCs at 85% confluency, the medium was changed to heparin medium (DMEM-F12 50/50(Thermo Fisher Scientific, 31331) supplemented with 213 μg/ mL L-ascorbic acid 2 phosphate (Sigma-Aldrich, A8960–5G), 1:100 Chemically defined lipid concentrate (Thermo Fisher Scientific, 11905031), 1.5 IU/mL Heparin (Leo Pharmaceuticals Ltd.), 1% Penicillin-Streptomycin (Gibco, 15-140-122)) supplemented with 4 × 10^–6^ M CHIR99021 (Selleck Chemicals). On day 2, the medium was replaced with a heparin medium containing 2 × 10^–6^ M Wnt-C59 (Tocris Bioscience). On day 4 and 6, the medium was replaced with heparin medium. From day 7, the medium was changed every other day with insulin medium (DMEM-F12 50/50 (Thermo Fisher Scientific, 31331) supplemented with 213 μg/ mL L-ascorbic acid 2 phosphate (Sigma-Aldrich, A8960-5G), 1:100 Chemically defined lipid concentrate (Thermo Fisher Scientific, 11905031), 21 μg/mL Human recombinant insulin (Sigma-Aldrich, I9278-5 ML), 1% penicillin/streptomycin (Gibco, 15-140-122)) until purification at day 10. iPSC-CMs started beating around day 10, then the medium was changed to purification medium (RPMI 1640 L-Glutamine without glucose (Gibco, 11879), supplemented with 3.5 μM Sodium-dl-Lactate (Sigma-Aldrich, L4263), 213 μg/mL L-ascorbic acid 2 phosphate (Sigma-Aldrich, A8960-5G), 1:100 Chemically defined lipid concentrate (Thermo Fisher Scientific 11905031), 21 μg/mL Human recombinant insulin (Sigma-Aldrich I9278-5 ML), 1% penicillin/streptomycin (Gibco 15-140-122) until day 15 after which the medium was changed to insulin medium, refreshed every other day. Cell cultures were tested negative for mycoplasma contamination using MycoAlert Kit (Lonza).

### Human iPSC-CM Characterization

#### Immunostaining Protocol

iPSC-CMs were fixated using 4% paraformaldehyde, permeabilized using 0.1% Triton-X-100 (Sigma-Aldrich) for 10 min, blocked with 10% normal goat serum (Sigma-Aldrich) for 30 min, and incubated at 4 °C overnight with primary antibodies (1:300 Mouse anti-α-actinin, Merck A7811, and 1:200 Rabbit antivimentin ab92547) diluted in DPBS. Fluorescent labeling was achieved using goat antimouse Alexa fluor-488, and goat antirabbit Alexa fluor-568 antibodies (Thermo Fisher Scientific, 1:500), and nuclear labeling using 1 μg/mL Hoechst (Thermo Fisher Scientific) for 1 h at room temperature. Imaging was performed by using a Leica SP8X confocal microscope.

#### Flow Cytometry

iPSC-CMs were detached using TrypLE select 10x (Thermo Fisher, A12177) for 10 min, centrifuged for 3 min at 200*g*, washed with PBS, and resuspended in permeabilization buffer containing 5% BSA and 0.3% Triton X-100 for 30 min. Cells were incubated with mouse anti-α-actinin (1:300 Merck A7811) or isotype control antibodies (FITC mouse IgM, κ isotype (1:200 dilution)) in flow cytometry buffer for 30 min at 4 °C. Cells were then washed three times with flow cytometry buffer, stained using Goat-antimouse (1:300 dilution) secondary-antibody, and analyzed using Cytoflex flow cytometer (Beckman Coulter).

### *In Vitro* Transfection

#### Transfection of HeLa and Jurkat Cells

HeLa cells were seeded in an 8-well confocal plate at a density of 5 × 10^4^ cells/well for confocal microscopic imaging (Leica TCS SP8 confocal laser scanning microscope). HeLa and/or Jurkat cells were seeded in 96-well plates at a density of 1 × 10^4^ cells/well for flow cytometry measurements (CytoFLEX S, Beckman Coulter). For the 2-step incubation protocol: cells were pretreated with CPK4 (10 μM) diluted in cell culture medium and cultured for 2 h, then replaced by medium containing LNP-CPE4 (1 μg/mL EGFP-mRNA) and incubated for 24 h before measuring. For the 1-step incubation protocol: a medium containing CPK4/K4 and LNP-CPE4 (1 μg/mL EGFP-mRNA, CPE4 = 2.5 μM, CPK4/K4 = 2.5 μM) was added to cells and incubated for 24 h before measuring.

To determine the optimal CPK4:CPE4 ratio for mRNA transfection, HeLa cells were seeded in a 96-well plate at a density of 1 × 10^4^ cells/well. The concentration of EGFP-mRNA in LNPs added to cells was 1 μg/mL. CPK4 and LNP1-CPE4 with different final ratios of CPK4:CPE4 (CPK4:CPE4 = 8:1, 4:1, 3:1, 2:1, 1:1, 0.5:1, 0.2:1, 0.1:1) were added together to cells and incubated for 24 h and analyzed by flow cytometry.

#### Transfection of iPSC-CMs

iPSC-CMs were seeded at the density of 1*10^5^ cells/well in a 96-well plate for flow cytometry measurements (BD FACSCanto, BD Biosciences) or at the density of 5*10^5^ cells/well in a 24-well confocal plate for confocal imaging. For the two-step 24h incubation protocol: iPSC-CMs cells were pretreated with CPK4 (10 μM) for 2 h. Next, the medium was replaced by LNP-CPE4 (EGFP-mRNA, 2 μg/mL) and incubated for 24 h before imaging and flow cytometry measurements. For the 1-step 24h incubation protocol: medium containing CPK4 and LNPs (EGFP-mRNA, 2 μg/mL, CPE4 = 5 μM, CPK4 = 5 μM) was added to the iPSC-CMs and incubated for 24 h before imaging and flow cytometry measurements. For the 2-step 2h and 1-step 2h incubation groups, the same procedures were followed, and LNPs were incubated with cells for 2 h, then replaced with fresh medium, and incubated for another 24 h before imaging and flow cytometry measurements. Lipofectamine MessengerMAX was used as positive control following the standard protocol.

### Cell Uptake Mechanism Study

iPSC-CMs were seeded in a 96-well plate at adensity of 1 × 10^5^ cells/well. iPSC-CMs were pretreated with dynasore (80 μM) for 2 h. Then, DiD labelled LNPs were added to the cells in the presence of fresh inhibitors or incubated at 4 °C (2 μg/mL, 2 h). Next, the cells were washed, dettached, and analysed by flow cytometry (BD FACSCanto, BD Biosciences). 0.5 mol% of DiD was included in the lipid composition and the DiD intensity was normalized to LNPs in the absence of inhibitors.

### Animal Experiments

#### Ethical Statement on Animal Experiments

All animal experiments were performed with the authorization of the Utrecht Animal Welfare Body and complied with the Dutch Experiments on Animals Act (WOD) under a license (AVD115002015257). The experiments were carried out following the Guide for the Care and Use of Laboratory Animals. The animals used in these experiments received water and standard chow *ad libitum* and were housed under standard conditions with 12 h light/dark cycles. Animals were randomized between the different treatment groups, and all operators were blinded during the experimental procedures.

#### Intravenous Injections

Male BALB/cByJ mice (Charles River Laboratories, 25–30 g, 12–13 weeks) were intravenously injected with 5 μg (in 100 μL) of LNP-encapsulated firefly luciferase mRNA via the tail vein (*n* = 4 per treatment). As background control, mice injected with PBS (*n* = 1) were used. Before LNP injection, for the CPK4/LNP1-CPE4 treatment, both CPK4 and LNP1-CPE4 were premixed in a ratio of 1:1 (final CPK4: 12.5 μM; Luc-mRNA: 50 μg/mL).

#### Intramyocardial Injections

To perform the intramyocardial injections, mice were anesthetized with an intraperitoneal injection of fentanyl (0.05 mg/kg of body weight), midazolam (5 mg/kg of body weight), and medetomidine (0.5 mg/kg of body weight), followed by intubation and connection to a respirator with a 1:1 oxygen/air ratio (times/min). During surgery, the body temperature was maintained at 37 °C by using a heating pad. To access the heart, a left lateral thoracotomy was performed, and 25G needles were used. Around 3 mm of the needle tip was introduced into the left ventricular wall, and a volume of 10 μL was injected at a rate of 10 μL per minute using a remote infuse/withdraw syringe pump (Pump 11 Elite Nanomite) loaded with a 25 μL Hamilton syringe (model 1702 RN SYR). At last, the surgical wounds were closed, and the mice were subcutaneously injected with an agonist consisting of atipamezole hydrochloride (3.3 mg/kg weight), flumazenil (0.5 mg/kg body weight), and buprenorphine (0.15 mg/kg body weight) for pain relief. Male BALB/cByJ mice (Charles River Laboratories, 25–30 g, 12–13 weeks) were intramyocardially injected with 10 μL containing 5 μg of LNP-encapsulated firefly luciferase mRNA (n = 6 per treatment). As controls, free mRNA and PBS were used (n = 3 and n = 2 respectively). For the one-step treatment, CPK and LNP1-CPE4 were premixed in a ratio of 1:1 (final CPK4: 125 μM, Luc-mRNA:500 μg/mL) before injection. For the two-step treatment, 5 μL of CPK4 (250 μM) was first injected, and after 10 min, 5 μL of LNP1-CPE4 (Luc-mRNA: 1000 μg/mL) were injected. For both 1-step and 2-step injection of coiled-coil modified LNPs, 2 replicates were found dead before performing the readouts. Moreover, 1 animal of the LNP1 group died during the injection.

#### Bioluminescence and Tissue Collection

Twenty-four hours after injection, the mice were intraperitoneally injected with 100 μL of D-luciferin (Promega) at a concentration of 25 mg/mL in DPBS (Dulbecco’s Phosphate Buffered Saline, Sigma). After 15 min, the mice were sacrificed, and the heart, lungs, liver, spleen and kidneys were collected. The luminescence of the organs was analyzed using an *in vivo* imaging system (IVIS, RT PhotonImager, BioSpace Lab) and quantified using the M3 Vision Software (BioSpace Lab). After imaging, organs were snap-frozen in liquid nitrogen and stored at −80 °C until further analysis. Blood was collected by cheek puncture in serum separation tubes (Sarstedt AG & Co. KG). The serum was separated by centrifugation (10 000*g*, 5 min, 4 °C) and stored at −80 °C until further analysis.

#### *Ex Vivo* Assessment of Luciferase Activity in Mouse Organs

Organ pieces of 50–200 mg were weighed and added to a tube containing a layer of 4–5 mm of beads (Qiagen). Per milligram of tissue, 5 μL of 1x Cell Culture Lysis Reagent (Promega) with 100x diluted Protease/Phosphatase inhibitor cocktail (Cell Signaling Technology) was added. Afterwards, the tissues were homogenized for 60 s using a Mini bead-beater (Bertin Technologies) at 5000 rpm. Samples were centrifuged for 10 min at 10 000 g and 4 °C. The supernatant was transferred to another tube, and finally, the luciferase activity was measured using a Spectramax ID3 with an injector (Molecular Devices). For that, 10 μL of supernatant was added to a white 96-well plate (Greiner) and 50 μL of Luciferase assay reagent (Promega) was dispensed using the injector. Then, the reaction was incubated for 2 s, and the luminescence was recorded and integrated over 10 s.

#### *In Vivo* Toxicity Evaluation

To assess liver function and treatment-induced toxicity, the levels of ALT, AST, and ALP from serum samples were assessed using ELISA kits (Abebio, Abcam, and FineTest respectively).

### Statistical Analysis

All experiments were performed at least in triplicate (*n* = 3) unless specified otherwise, and the significance was determined using an unpaired student *t* test or one-way ANOVA (GraphPad Prism) for all comparisons. ****, *P* < 0.0001, ***, *P* < 0.001, **, *P* < 0.01, *, *P* < 0.05, ns, no significant difference.

## Supporting Information Available

Structure of the lipids and lipopeptides; lipid compositions of LNP; physicochemical characterization of the LNP formulations; hydrodynamic diameter changes of LNP1-CPE4; Temperature dependence of the steady-state fluorescence anisotropy rss of TMA-DPH assay in LNPs; mRNA transfection efficiency of HeLa cells after incubation with LNPs; mRNA transfection efficiency in iPSC-CMs of LNPs modified with fusogenic coiled-coil peptides; intravenous administration of coiled-coil peptide modified mRNA-LNPs; intramyocardial injection of coiled-coil fusogenic peptides modified LNP-mediated mRNA delivery; safety evaluation of LNPs; cell uptake of plain LNP1 by iPSC-CMs (PDF)
