# Supplementary material for: Fusogenic Coiled-Coil Peptides Enhance Lipid Nanoparticle-Mediated mRNA Delivery upon Intramyocardial Administration
Source: ACS Nano. 2023 Nov 20;17(23):23466–77. doi: 10.1021/acsnano.3c05341 (PMC10722601; doi:10.1021/acsnano.3c05341)
Supplement: Supplementary file 1 — nn3c05341_si_001.pdf [file nn3c05341_si_001.pdf]

Supporting Information

of

Fusogenic coiled-coil peptides enhance lipid nanoparticle-mediated mRNA delivery upon intramyocardial administration

Ye Zeng[1#], Mariona Estapé Senti[2#], M. Clara I. Labonia[3], Panagiota Papadopoulou[1], Maike A.D. Brans[3], Inge Dokter[3,4], Marcel H. Fens[5], Alain van Mil[3,4], Joost P.G. Sluijter[3,4], Raymond M. Schiffelers[2], Pieter Vader[2,3], and Alexander Kros[1]**

[1] Department of Supramolecular & Biomaterials Chemistry, Leiden Institute of Chemistry, Leiden University, 2333 CC Leiden, The Netherlands.

[2] CDL Research, University Medical Center Utrecht, 3584 CX Utrecht, The Netherlands

[3] Department of Cardiology, Laboratory of Experimental Cardiology, University Medical Center Utrecht, 3584 CX Utrecht, The Netherlands

[4] Regenerative Medicine Center Utrecht, University Utrecht, University Medical Center Utrecht, 3584 CX Utrecht, The Netherlands

[5] Department of Pharmaceutics, Utrecht Institute for Pharmaceutical Sciences, Utrecht University, 3584 CX Utrecht, The Netherlands

# These authors contributed equally: Ye Zeng, Mariona Estape Senti.

*Correspondence:

Pieter Vader, CDL Research, University Medical Center Utrecht, Heidelberglaan 100, 3584 CX Utrecht, The Netherlands, E-mail: pvader2@umcutrecht.nl;

Alexander Kros, Leiden Institute of Chemistry, Leiden University, Einsteinweg 55, 2333 CC Leiden, The Netherlands, E-mail: a.kros@chem.leidenuniv.nl

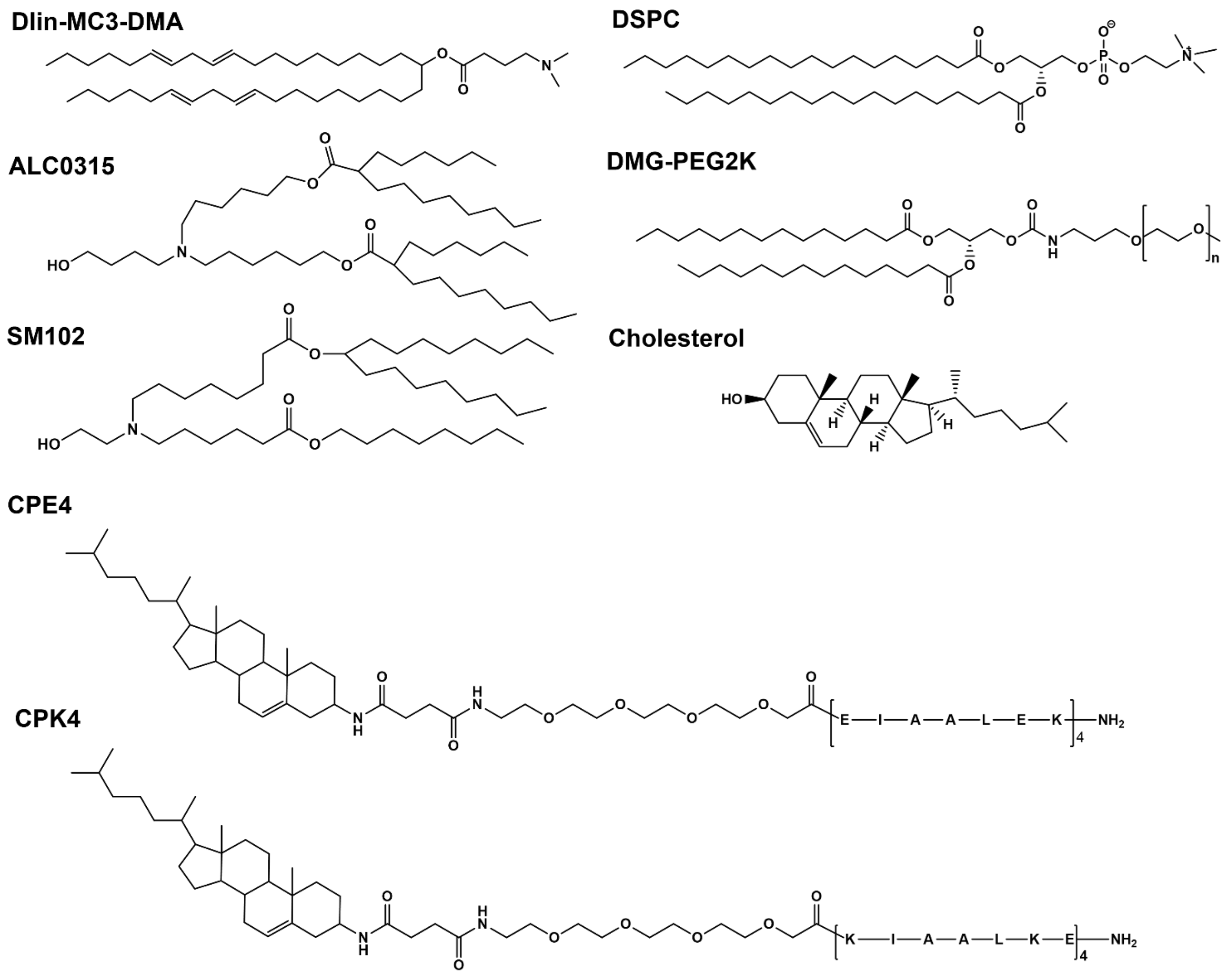

Scheme S1. Structure of the lipids and lipopeptides used in this study.

Table S1. Lipid compositions of LNP (mol%).

	LNP1	LNP1-CPE4	LNP2	LNP2-CPE4	LNP3	LNP3-CPE4
**Dlin-MC3-DMA**	50	50	-	-	-	-
**ALC-0315**	-	-	50	50	-	-
**SM-102**	-	-	-	-	50	50
**cholesterol**	38.5	37.5	38.5	37.5	38.5	37.5
**DSPC**	10	10	10	10	10	10
**DMG-PEG2K**	1.5	1.5	1.5	1.5	1.5	1.5
**CPE4**	-	1	-	1	-	1

Table S2. Physicochemical characterization of the LNP formulations used in this study.

	**Hydrodynamic diameter (nm)**	**PDI**	**Zeta-potential (mV)**	**Encapsulation efficiency (%)**
**LNP1**	83.6±2.8	0.112±0.02	-3.38±0.42	92.34±2.41
**LNP1-CPE4**	92.1±1.3	0.134±0.05	-4.24±0.74	82.60±4.25
**LNP2**	86.6±3.8	0.132±0.024	-4.01±0.66	90.20±2.71
**LNP2-CPE4**	98.5±2.3	0.154±0.05	-7.29±0.88	81.15±5.35
**LNP3**	95.5±2.7	0.172±0.024	-5.36±0.61	89.54±2.35
**LNP3-CPE4**	116.2±1.4	0.134±0.057	-8.13±0.68	82.48±3.47

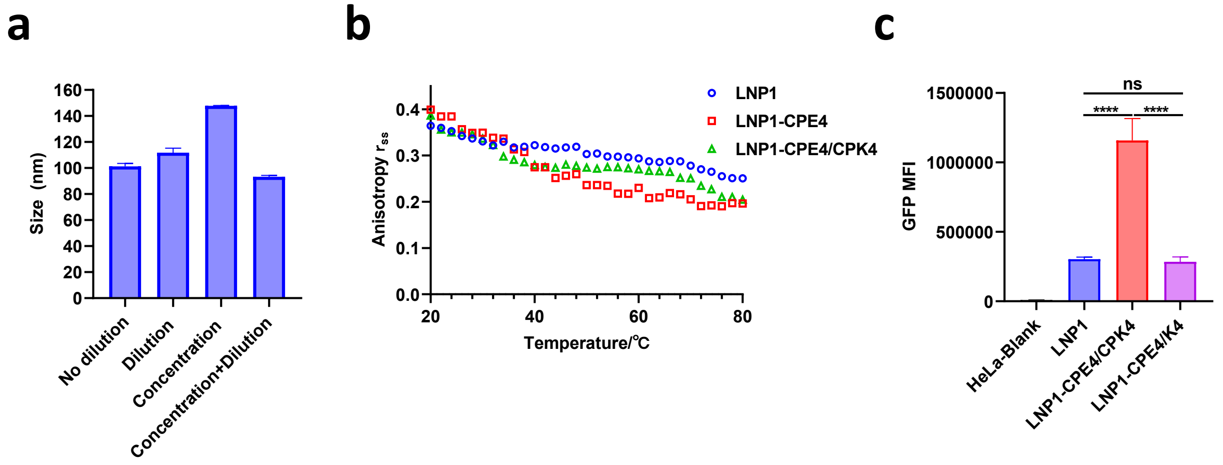

Figure S1. (a) Hydrodynamic diameter changes of LNP1-CPE4 as a result of dilution and concentration of the sample. No dilution: freshly prepared LNP1-CPE4; Dilution: 10x dilution of freshly prepared LNP1-CPE4; Concentration: freshly prepared LNP1-CPE4 concentrated (30x) to reach intramyocardial injection concentration (500 µg/mL mRNA); Concentration+Dilution: concentrated LNP1-CPE4 then 60x dilution. Data are represented as the mean ± s.d. (n=3) (b) Temperature dependence of the steady-state fluorescence anisotropy r_{ss} of TMA-DPH assay in LNPs. (c) Flow cytometry measurements of GFP expression intensity (GFP MFI) of HeLa cells after incubation with LNPs encapsulating EGFP-mRNA (mean ± s.d., n=3, ****, $P < 0.0001$, ***, $P < 0.001$, **, $P < 0.01$, *, $P < 0.05$, ns, no significant difference).

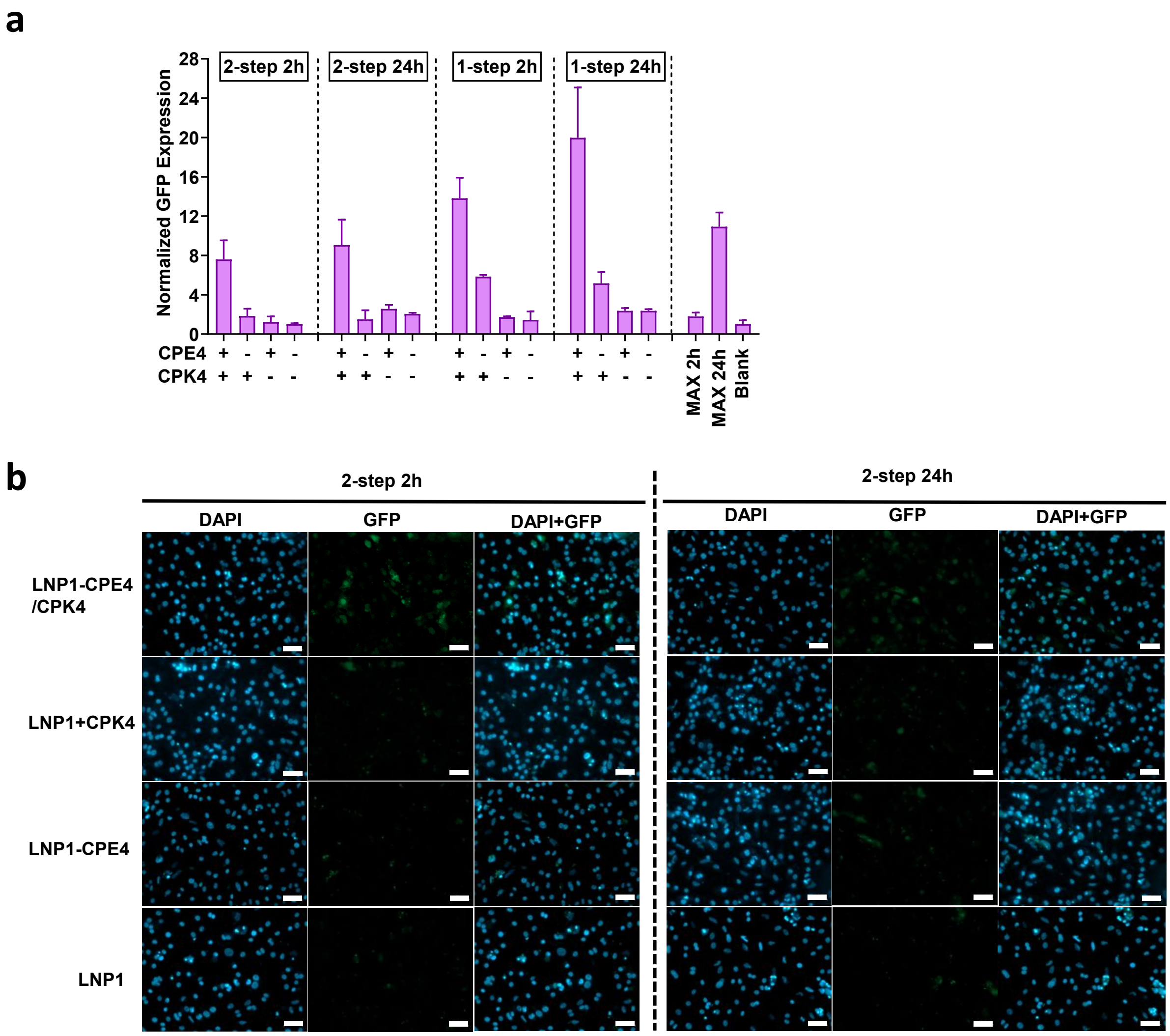

Figure S2. mRNA transfection efficiency in iPSC-CMs of LNPs modified with fusogenic coiled-coil peptides. (a) Flow cytometry analysis of transfection efficiency enhancement of LNPs in iPSC-CMs with GFP intensity (GFP MFI) normalized to LNP1 (2-step incubation, 2 h). (b) Confocal microscopy images of the EGFP-mRNA (2 µg/mL) transfection of LNPs in iPSC-CM using a 2-step incubation protocol. Blue: DAPI; green: GFP, green fluorescent protein; scale bar is 50 µm.

a

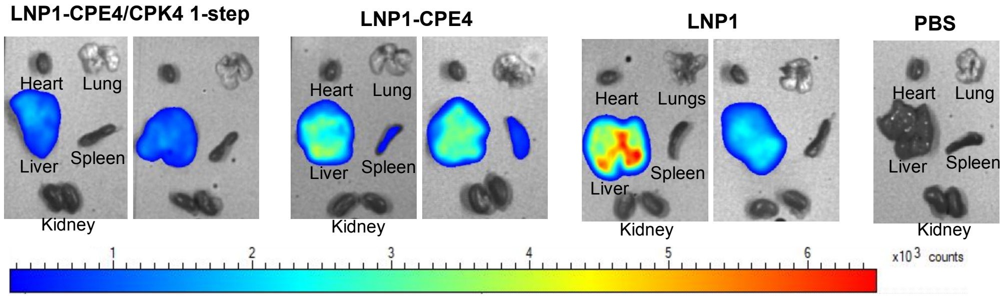

b

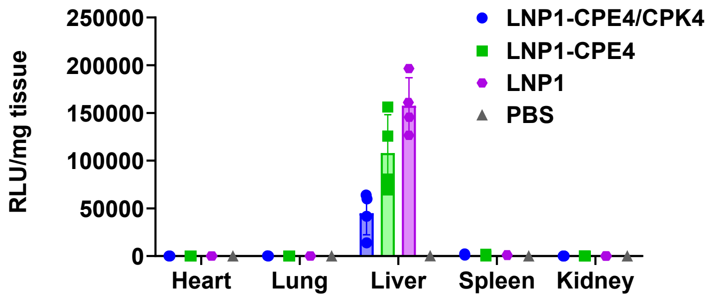

Figure S3. Intravenous administration of coiled-coil peptide modified mRNA-LNPs did not result in delivery to the heart. Balb/c mice were intravenously injected with 5 μg (in 100 μL) of firefly luciferase mRNA encapsulated in (1) CPK4 and LNP1-CPE4 prepared using the 1-step protocol, (2) LNP1-CPE4 or (3) LNP1. A control injection of PBS only was used to determine the background level. Organs were harvested 24 h after administration and the luminescence was measured by IVIS imaging. (a) Luminescence images of mice organs. (b) Luciferase activity in organ lysates. Data are represented as the mean ± s.d. (n=4 biologically independent mice per group; PBS: n=1.)

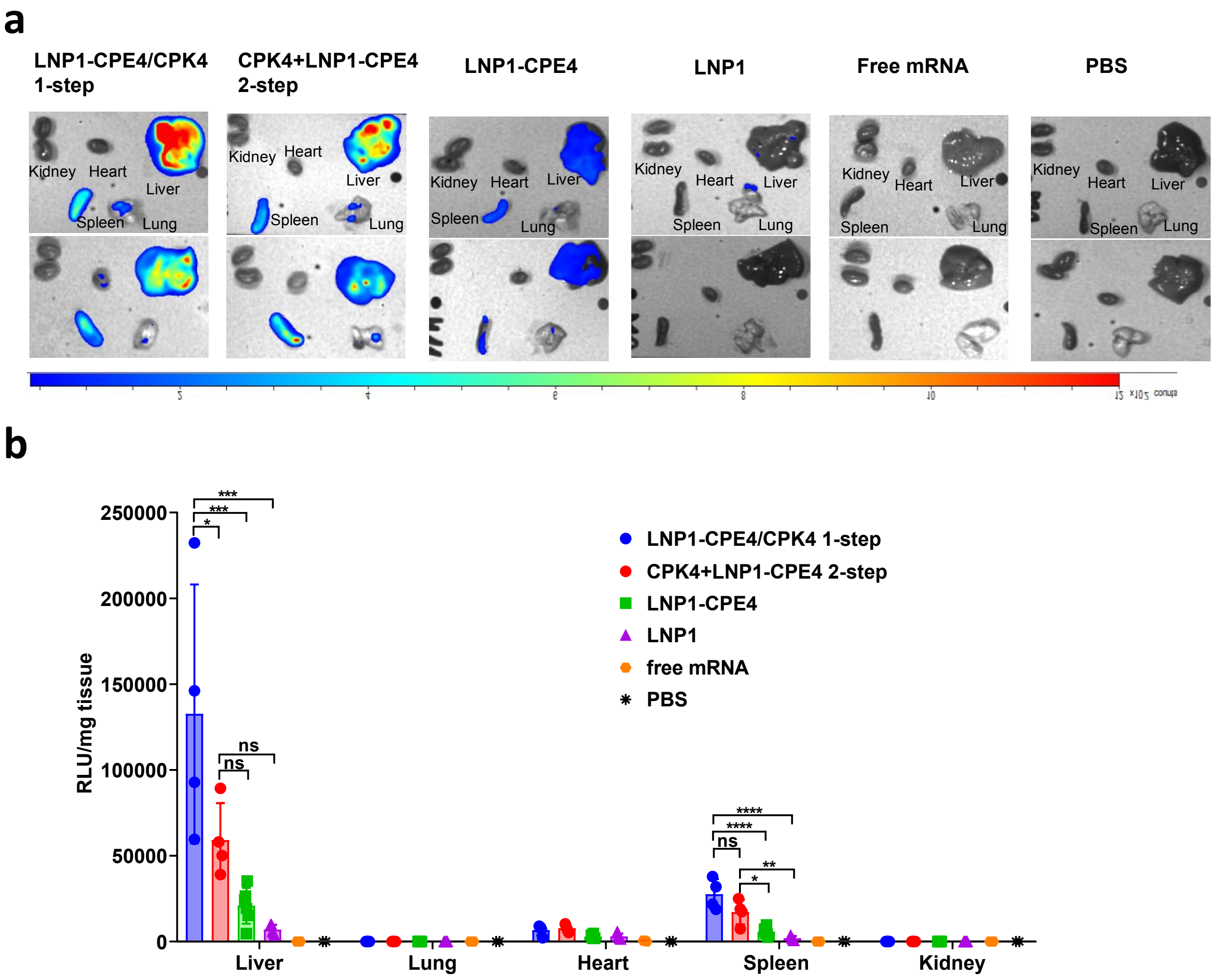

Figure S4. Intramyocardial injection of coiled-coil fusogenic peptides modified LNP-mediated mRNA delivery. (a) Luminescence images of mice organs of LNPs upon intramyocardial injection. (b) Luciferase activity in organ lysates. Statistical significance was calculated with a One-Way ANOVA (****, P < 0.0001, ***, P < 0.001, **, P < 0.01, *, P < 0.05, ns, no significant difference). Data are represented as the mean ± s.d. (n=4 for LNP1-CPE4/CPK4 1-step and CPK4+LNP1-CPE4 2-step; n=6 for LNP1-CPE4; n=5 for LNP1; n=3 for free mRNA; n=2 for PBS.)

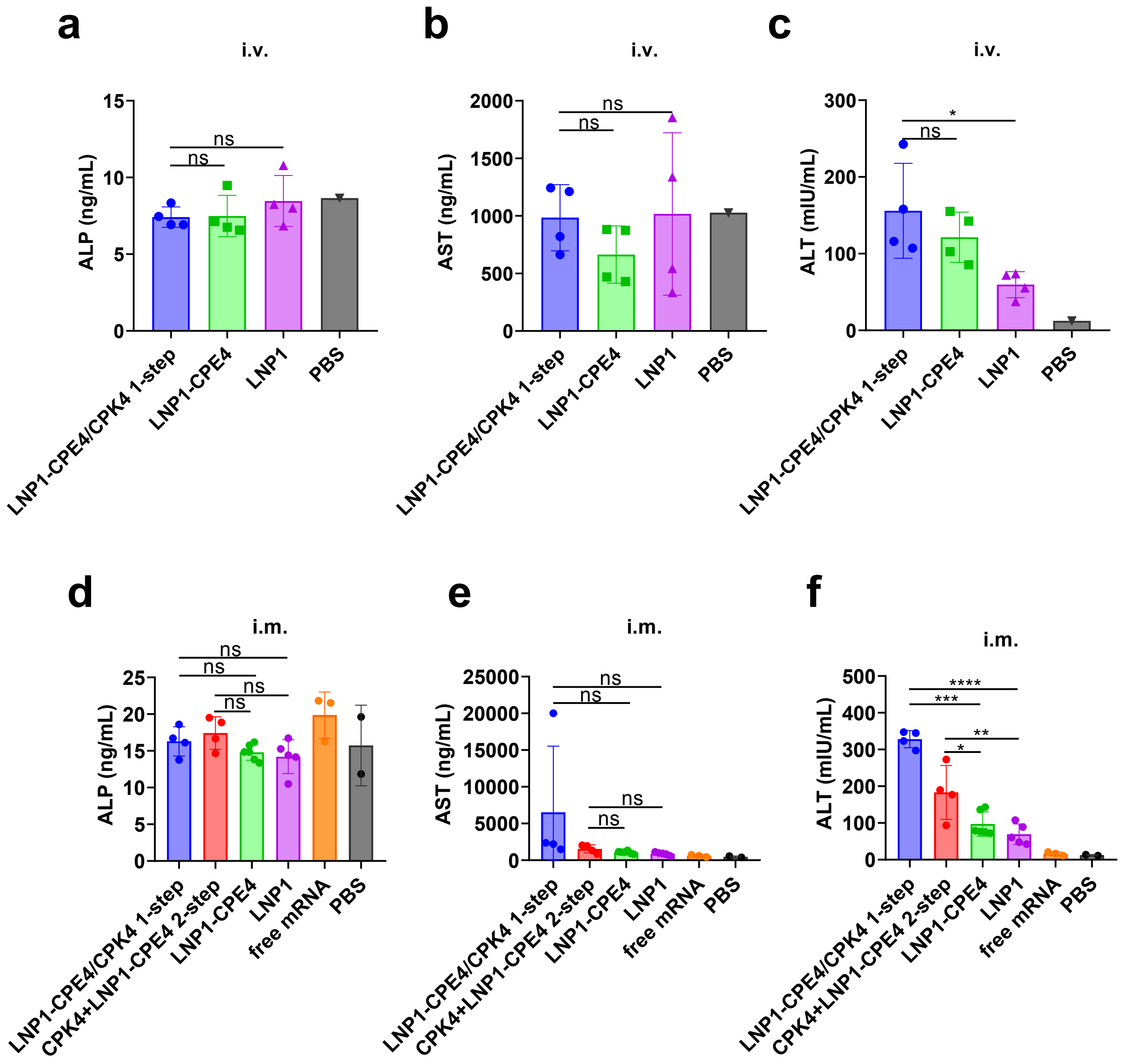

Figure S5. Safety evaluation of LNPs. (a-c) Serum levels of liver enzymes were analyzed to determine the safety profile of LNPs after intravenous injections. (a) Alkaline phosphatase (ALP), (b) Aspartate transaminase (AST), and (c) Alanine aminotransferase (ALT). Data are represented as the mean ± s.d. (n=4 biologically independent mice per group; PBS: n=1.) (d-f) Serum levels of liver enzymes were analyzed to determine the safety profile of LNPs after intramyocardial injections (d) ALP, (e) AST, and (f) ALT. Data are represented as the mean ± s.d. (n=4 for LNP1-CPE4/CPK4 1-step and CPK4+LNP1-CPE4 2-step; n=6 for LNP1-CPE4; n=5

for LNP1; n=3 for free mRNA; n=2 for PBS.) Statistical significance was calculated with a One-Way ANOVA (****, P < 0.0001, ***, P < 0.001, **, P < 0.01, *, P < 0.05, ns, no significant difference).

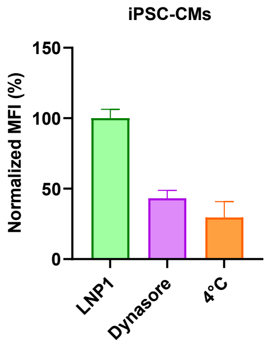

Figure S6. Cell uptake of plain LNP1 by iPSC-CMs after 2 h incubation in the presence of Dynasore or incubated at 4 °C. 0.5 mol% of DiD was included in the lipid composition and DiD intensity was normalized to =LNP1 uptake in the absence of inhibitor. Error bars represent mean ± s.d. (n=3).
